# Valvular Lesions of the Heart

**Published:** 1880-09

**Authors:** 


VALVULAR LESIONS OF THE HEART.

Dr. Austin Flint concludes a clinical lecture on this subject, summarizing the practical points as follows:

1. Cardiac murmurs may represent lesions which, if unaccompanied by symptoms referable thereto, enlargement of the heart not co-existing, and the heart-sounds normal, are to be considered as innocuous. The prediction of grave consequences, under these circumstances, is unwarrantable, inasmuch as they may never occur. Such lesions do not claim medical treatment, nor any extraordinary precautions; and it is desirable that the fact of their existence be withheld from patients, if this can be done with propriety.

2. Patients with valvular lesions are liable to suffer from functional disorders of the heart, arising from causes which have no pathological connection with the lesions. It is highly important to recognize, clinically, this accidental coincidence, in order to exercise a correct judgment as to the prognosis and treatment.

3. Various morbid conditions, other than functional disorder of the heart, may be accidentally associated with valvular lesions and more or less cardiac enlargement. These associated morbid conditions may be, in a great measure, responsible for symptoms and effects which seem to denote an advanced stage of the cardiac disease, whereas, the latter may occasion but little inconvenience, provided these accessory, co-operating conditions can be removed.

4. Valvular lesions involving either obstruction or regurgitation, or both combined, and having led to considerable or even great enlargement of the heart, under favorable circumstances as regards associated morbid conditions, are often well tolerated indefinitely. There is less reason for a hopeful prognosis, in respect of tolerance, when there is considerable aortic insufficiency, than in cases of aortic obstructive lesions, and those which occasion obstruction or regurgitation at the mitral orifice. The danger of sudden death from aortic regurgitation is lessened by co-exisiting mitral insufficiency.

5. In cases of orthopnœa and general dropsy dependent on mitral obstructive or regurgitant lesions and enlargement of the heart, digitalis and active hydragogue purgation repeated from time to time, not only afford notable relief, but there is reason to believe that life is sometimes thereby much prolonged.—*Medical News and Abstract*, Jan., 1880.

